# Examination of food reward and energy intake under laboratory and free-living conditions in a trait binge eating subtype of obesity

**DOI:** 10.3389/fpsyg.2013.00757

**Published:** 2013-10-21

**Authors:** Michelle Dalton, John Blundell, Graham S. Finlayson

**Affiliations:** Human Appetite Research Unit, Faculty of Medicine and Health, Institute of Psychological Sciences, University of LeedsLeeds, UK

**Keywords:** hedonic eating, food reward, binge eating trait, implicit wanting, explicit liking, dietary recall

## Abstract

**Background/Aims:** Trait binge eating has been proposed as a “hedonic subtype” of obesity characterized by enhanced food liking and wanting, and a preference for high-fat sweet foods in the laboratory. The current study examined the influence of trait binge eating in overweight or obese women on eating behavior under laboratory and free-living conditions over a 48-h period.

**Methods:** In a matched pairs design, 24 overweight or obese females (BMI: 30.30 ± 2.60 kg/m^2^; Age: 25.42 ± 3.65 years) with high or low scores on the Binge Eating Scale (BSE) were divided into one of two groups; Obese Binge (O-B) and Obese Non-binge (O-NB). Energy intake was assessed using combined laboratory energy intake measures and 24-h dietary recall procedures. Liking and wanting were assessed using the Leeds Food Preference Questionnaire (LFPQ).

**Results:** There was a significant association between overall energy consumed, and energy consumed from snack foods under laboratory and free-living conditions. O-B exhibited a greater preference for sweet snack foods in their laboratory and free-living eating behavior. These findings were supported by greater laboratory-based measures of wanting and craving for this food type in O-B. In addition, O-B consumed significantly more energy than their estimated daily energy requirements in the laboratory suggesting that they over-consumed compared to O-NB.

**Conclusions:** The measurement concordance between laboratory and free-living based energy intake supports the validity of laboratory-based test meal methodologies Variation in trait binge eating was associated with increased craving and wanting for high-fat sweet foods and overconsumption in the laboratory. These findings support the use of trait binge eating as a common hedonic subtype of obesity and extend the relevance of this subtype to habitual patterns of energy intake.

## Introduction

In the recent publication of the Diagnostic and Statistical Manual of Mental Disorders (DSM-5) Binge Eating Disorder (BED) has been formally recognized as an eating disorder. The prevalence of BED in the general population is estimated to be between 0.7 and 3.0% and is commonly comorbid with overweight and obesity (Brownley et al., 2007; Kessler et al., 2013). However, the emotions, cognitions, and behaviors associated with the tendency to binge eat, are estimated to occur in 10–20% of the general obese and lean population (Spitzer et al., 1993; Berg et al., 2009; Striegel-Moore et al., 2009) and together constitute a trait that can be psychometrically assessed along a continuum and applied to the general population. Importantly, this trait or disposition to binge eat, assessed by the Binge Eating Scale (BES; Gormally et al., 1982) appears to be functional at low to moderate levels in lean and overweight or obese females (Finlayson et al., 2011; Dalton et al., 2013) and has been proposed as a plausible subtype of obesity (Hudson et al., 2006; Davis et al., 2009; Davis, 2013).

In a study of non-obese females who varied in their scores on the BES, those with high scores selected and consumed a greater quantity of high-fat sweet foods, compared to those with low scores. Moreover, those with high scores displayed greater wanting for high-fat sweet foods, as assessed by a computer-based behavioral task (Finlayson et al., 2011). In an extension to these findings, we subsequently examined the influence of trait binge eating on food reward and energy intake under fed and fasted states, in age-matched lean and overweight or obese females (Dalton et al., 2013). As previously, we found that those with high scores were characterized by an increased intake of high-fat sweet foods and enhanced wanting for these foods compared to those with low scores; and that these differences were more pronounced when fasted. Taken together, these findings suggest that the disposition to binge eat (termed here “trait binge eating”) is a functionally meaningful individual difference metric and can be used to identify a behavioral subtype of the population who are susceptible to overeating. Furthermore, under laboratory conditions these susceptible individuals are characterized by a behavioral profile of enhanced wanting for high-fat sweet foods compared to age and BMI-matched controls.

While the assessment of trait binge eating behavior in the laboratory allows for increased levels of experimental control over the measurement of food preference and energy intake, through the use of laboratory techniques alone it is unknown whether the eating behavior observed will generalize to free-living eating behavior. Research utilizing food diaries and food recall procedures allow for the study of eating behavior and dietary patterns in the natural setting. However, relatively few studies have attempted to combine laboratory eating behavior measures with free-living eating behavior measures in order to examine the ecological validity of overeating observed in the laboratory setting.

Therefore, the present study had two primary aims: (1) to assess the concordance between laboratory-based and free-living based measures of eating behavior, (2) to examine the effect of trait binge eating in a sample of overweight or obese females on energy intake, food choice, food reward, and food cravings during two 24-h periods: firstly using test meal methodology in the laboratory; and secondly using a validated dietary recall technique, in the natural, unrestricted setting. We hypothesized that greater levels of binge eating would be associated with greater energy intake and a preference for high-fat sweet foods both in the laboratory and in the participants' natural setting. Furthermore, in line with previous findings, we hypothesized that “binge-types” would have greater liking and wanting for high-fat sweet foods compared to “non-binge types.”

## Methods

### Participants

Twenty-four females were included in the final study after an initial screening and experimental group allocation process involving a sample of 137 volunteers. Participants were overweight or obese (age: 25.42 ± 6.42, BMI: 30.30 ± 2.60) females recruited from the staff and student population at the University of Leeds. The initial screening process excluded those who were taking medication, currently dieting, reported a history of eating disorders, or were unfamiliar with or disliked any of the study foods. In addition, participants were screened on the basis of their scores on the Binge Eating Scale (BES; Gormally et al., 1982). Accordingly, twelve individuals scoring ≥ 18 formed the “obese binge type” (O-B; age: 25.67 ± 6.28, BMI: 31.48 ± 2.65) and twelve individuals scoring ≤ 6 formed the “obese non-binge type” (O-NB; age: 25.17 ± 5.75, BMI: 30.12 ± 1.55) groups. The groups were individually matched by age and BMI, with an overall difference of no more than 3 years and 3 BMI points across each matched pair. Informed written consent was obtained prior to the study. Participants received £15 for their participation. All research procedures were reviewed and approved by the University of Leeds, Institute of Psychological Sciences in accordance with the Helsinki declaration.

### Design and procedure

A between subjects (O-B or O-NB) design was used with participants attending the research unit on two occasions over the course of 3 days. Test sessions were held in the follicular phase of the participants' menstrual cycle in order to reduce the influence that the luteal phase may have on energy intake and food choice (Cohen et al., 1987; Dye and Blundell, 1997). In the first visit (Day 1), energy intake at breakfast, lunch, and dinner was assessed using test meal methodology in the laboratory and the test meals were designed to capture participants' usual energy intake. Each eating occasion was separated by a period of 4 h, during which the participants were allowed to leave the research unit. While outside of the research unit participants were instructed not to eat or drink any food or beverages, except water, unless provided by the researcher. In the 4-h period between lunch and dinner, participants were provided with a snack box to measure snack food intake. To ensure that participants arrived at the research unit in a standardized, fasted state they were instructed not to eat from 10 pm the evening before. Subjective ratings of appetite and mood were taken at the start of Day 1 and then at hourly intervals and following each event in the procedure.

The second visit was held on Day 3 of the procedure. During this visit free-living energy intake was assessed for the previous 24 h during which participants performed their usual eating behaviors in the free-living environment (Day 2) using the validated Automated Multiple Pass Method (Moshfegh et al., 2008). Importantly, the purpose of the Day 3 visit was not disclosed in order to avoid the possibility of participants intentionally (for the purposes of the study) monitoring, restricting or rehearsing their food intake during the recall period on Day 2. In addition, this visit was held on either a Wednesday or Thursday to avoid weekend fluctuations in energy intake (de Castro, 2010). To eliminate the possibility of experimenter bias during data collection and data entry, participants' group status was assigned during initial screening, after which data were anonymised and stored separately until the data was compiled for analysis.

### Measures

#### Trait binge eating

Participants completed the Binge Eating Scale (BES; Gormally et al., 1982) during initial online screening. The BES is comprised of sixteen items, eight describing the behavioral manifestations and eight describing the feelings and cognitions associated with binge eating. Each item consists of 3–4 descriptive statements that increase in severity (e.g., “I don't have any difficulty eating slowly in the proper manner” to “I have the habit of bolting down my food without really chewing it. When this happens I usually feel uncomfortably stuffed because I've eaten too much.”) Participants are required to select the statement from each of the sixteen items that is most descriptive of them. Scores are summed to produce a total score ranging from 0 to 46. Cut off points have previously been reported denoting mild (≤17), moderate (18–26), and severe (≥27) binge eating behavior (Marcus et al., 1988). The BES has been shown to have good internal validity, with a Cronbach's alpha of 0.89 (Freitas et al., 2006) and good test-retest reliability (Timmerman, 1999).

#### Control of eating questionnaire

Participants completed the Control of Eating Questionnaire (COEQ; Hill et al., 1991; Greenway et al., 2010) at the end of the study procedures. The COEQ is comprised of 21 items that are designed to assess the severity and type of food cravings experienced over the previous 7 days. The COEQ has five subscales; Craving Intensity, Craving for Savory foods, Craving for Sweet foods, Mood and Feelings of Fullness. Items on the COEQ are assessed by 100-mm visual analog scales (VAS) with items relating to each subscale being averaged to create a final score.

#### Anthropometrics and body composition

Standing height without shoes was measured to the nearest 0.5 cm using a stadiometer. Body weight was measured using an electronic balance and recorded to the nearest 0.1 kg. Waist circumference (cm) was measured 1 cm above the top of the participants' naval after expiration. Air plethysmography (Bodpod, Concord, CA, USA) was used in order to obtain an estimate of participant's fat mass, fat free mass, and percentage body fat. Measures of body composition were taken while wearing non-underwired swimwear and a swim cap. All measures were taken at the start of Day 1 following an overnight fast.

#### Subjective appetite and mood sensations

Subjective appetite and mood sensations were assessed using 100-mm VAS presented on the validated, hand-held Electronic Appetite Ratings System EARS II; HP iPAQ; (Gibbons et al., 2011). Measures of hunger (“how hungry do you feel now?”) and fullness (“how full do you feel right now?”) were anchored at each end with the statements “extremely” and “not at all.” Ratings of prospective consumption (“how much food could you eat right now?”) and desire to eat (“how strong is your desire to eat?”) were anchored at each end by “none at all” and “a very large amount” and “not very strong” and “very strong,” respectively. To assess mood participants were asked “How irritable do you feel right now?” and “How content do you feel right now?” with both questions being anchored with the statements “not at all” and “extremely.” These measures were taken at hourly intervals and after each event in the procedure during the first visit. Previous research has shown that VAS are sensitive to experimental manipulations and have good test-retest reliability (Stubbs et al., 2000).

#### Food reward: liking and wanting for food

The Leeds Food Preference Questionnaire (LFPQ) assessed liking and wanting for food. This behavioral task has been widely used in previous research (Griffioen-Roose et al., 2010; Finlayson et al., 2011) and is described in more detail elsewhere (Finlayson et al., 2011). The LFPQ assesses explicit liking and implicit wanting for food using photographic images of common food items. Stimuli are categorized according to fat content (high or low) and taste (sweet or non-sweet). To measure explicit liking, participants were required to rate “how pleasant would it be to taste some of this food now?” on a 100-mm VAS. The ratings for each food item were averaged for each food type. To measure implicit wanting, a forced choice methodology was used in which an image from a given type was paired against every other image over ninety-six trials. Participants were required to respond according to the prompt “which food do you most want to eat now?” as quickly and as accurately as possible and reaction times were covertly measured. This measure produces a covert, non-verbal indication of the implicit motivated preference (implicit wanting) of the food types assessed in the task (Finlayson et al., 2008, 2009) To increase statistical reliability and adjust for individual variability in overall time spent on the task, reaction times were transformed to a standardized “*d*-score” (D-RT) using a validated algorithm (Greenwald et al., 2003), the procedure for which is described in greater detail elsewhere (Finlayson et al., 2011). A greater implicit wanting score is indicative of a faster relative reaction time for that food type.

#### Estimation of daily energy requirements

Estimated daily energy requirements were calculated using the Schofield equations (Schofield, 1985) for basal metabolic rate multiplied by physical activity level (PAL) derived from self-reported frequency and mode of exercise performed per week. Estimated daily energy requirements were used to evaluate over- or under-consumption relative to energy needs.

#### Test foods

***Ad libitum test meals.*** Energy intake at breakfast, lunch, and dinner was examined in the research unit using test meal methodology. Participants ate alone in an experimental cubicle with water provided *ad libitum*. Prior to each meal they were instructed to eat until they were comfortably full. For the breakfast test meal, participants selected to receive one of two types of cereal, which was served alongside toast, milk, butter, and strawberry jam. For the lunch test meal, participants were provided with two types of sandwich, fruit yoghurt, and cheese savory crackers. Each sandwich was divided into quarters and to aid with the calculation of energy intake participants were informed, before they began eating, that they should finish any quarters they began to eat. For the dinner test meal, participants received pasta and pasta sauce with a side salad, garlic bread, and chocolate cake rolls. All foods were provided in *ad libitum* quantities and participants were provided with plates and bowls in order to allow them to serve themselves freely. The caloric content and weight of the food received for each eating occasion was as follows: Breakfast 1700 cal, 930 g; lunch 1600 cal, 700 g; dinner 2100 cal, 1525 g; snack box 1700 cal, 400 g. Participants were informed that they could request more of any of the items they received. When requested, tea and coffee were provided with each meal with optional milk and sugar. Each eating occasion was separated by a period of 4-h. Food was measured to the nearest 0.1 g and energy values were determined using food tables and manufacturer labeling.

***Ad libitum snack food choice and intake.*** Participants were given a snack box, which contained four pre-selected snack foods. Each snacks food represented one of the food categories presented in the LFPQ (high-fat savory, low-fat savory, high-fat sweet, and low-fat sweet). During screening, participants selected one item from a choice of three from each category by first ranking each snack food from “most preferred” to “least preferred” and then rating each item for pleasantness and frequency of consumption using seven-point Likert scales. Participants received 100 g of each item in clear plastic food bags, which were placed in a jute bag for them to take away and consume if and as they wished, in the 4-h period between lunch and dinner. Participants were told that they could consume as much or as little as they wanted of the items, but that they should not share, donate or dispose of them. The snack box was collected at the beginning of the dinner session. Food from the snack box was measured to the nearest 0.1 g and energy values were determined using manufacturer labeling.

***24-hour dietary recall.*** During Day 3 of the procedure, participants' free-living energy intake from Day 2 was measured via dietary recall using the Automated Multiple Pass Method (AMPM; Moshfegh et al., 2008). The AMPM consists of five stages. Briefly, the first stage included uninterrupted recall all of the food and beverage items consumed over the preceding 24-h period. For the second stage, the researcher cued recall of nine frequently forgotten food categories that included non-alcoholic and alcoholic beverages, fruit and bread items. For the third stage, each item reported was assigned to an eating occasion (e.g., breakfast, snack). For the fourth stage, detailed information was gathered about the items including brand names, portion size, and added items (e.g., condiments and fats). For the final stage, participants reviewed the items and were given the chance to recall any foods they may have missed, or report any small items of food they may not have felt was worth reporting. Measuring cups, spoons and images of food portions were provided to aid participants with the estimation of portion size. The total number of calories consumed was calculated for the recall based on manufacturers' labels and the nutrition analysis tool WinDiets.

### Data analyses

Data were analyzed using SPSS version 20 for Windows and are presented as means with standard error. Before analysis, the data were checked for normality and for outliers. To examine the influence of trait binge eating on appetite and mood ratings repeated measures ANOVA were conducted. Independent *t*-tests were used to examine the differences between binge-type groups. The effect of trait binge eating on energy intake from the snack foods during TM-EI and DR-EI was analyzed for overall energy intake, energy intake according to the taste of the snack foods (sweet or savory) and energy intake according to the taste and fat content (high or low) of the snack foods using a 2 × 2 and a 2 × 2 × 2 repeated measures ANOVA, respectively. Where appropriate, participants' fat-free mass and estimated energy requirements were added as covariates in the analysis. Food hedonics were analyzed according to motivational state (fasted or fed), fat content of food images (high-fat or low-fat), taste of food images (savory or sweet) and binge-type group using a 2 × 2 × 2 × 2 ANOVA. Where appropriate, Greenhouse-Geisser probability levels were used to adjust for non-sphericity. *Post hoc* analyses were conducted on significant interactions using the Bonferroni correction. An α-level of 0.05 was used to determine statistical significance.

## Results

### Sample characteristics

Group characteristics of age, BMI, body composition, and psychometric traits are shown in Table 1. According to design, trait binge eating score was greater in O-B compared to O-NB [*t*_(22)_ = 17.39, *p* < 0.001]. While there were no differences in BMI, O-B had greater fat mass than O-NB [*t*_(22)_ = 2.21, *p* < 0.05].

**Table 1:** **Characteristics (mean ± SEM) of age, anthropometrics, body composition, and psychometric traits for obese binge-type and obese non-binge type**.

	**Obese binge-type**	**Obese non-binge type**
Age	25.7 (2.1)	25.2 (1.7)
Height (cm)	169.3 (1.6)	164.9 (1.4)
Weight (kg)^*^	90.2 (4.0)	79.3 (1.7)
BMI (kg/m^2^)	31.5 (1.3)	30.1 (0.4)
Waist (cm)	98.1 (3.9)	90.6 (1.5)
Fat mass (kg)^*^	36.3 (3.8)	27.4 (1.4)
% Body fat	39.3 (2.5)	34.9 (1.6)
Fat free mass (kg)	53.9 (1.3)	50.2 (1.5)
Binge eating score^***^	21.1 (0.8)	5.0 (0.3)

* p < 0.05;

*** p < 0.001.

### Association between laboratory-based and free-living based measures of energy intake

Overall energy consumed under laboratory conditions was positively associated with overall energy consumed under free-living conditions [*r*_(24)_ = 0.585, *p* < 0.001]. Further to this, there was a positive association between energy consumed from snack foods under laboratory and free-living conditions [*r*_(24)_ = 0.434, *p* < 0.01]. When the effect of trait binge eating was considered, O-B consumed significantly more energy under laboratory compared to free-living conditions [*t*_(11)_ = 2.25, *p* < 0.05], while O-NB consumed a similar amount of energy under both measures of energy intake [*t*_(11)_ = 0.786, *p* = 0.45] (see Table 2).

**Table 2:** **Estimated 24-h energy requirements, laboratory total energy intake, and free-living total energy intake (mean ± SEM) for obese binge-type and obese non-binge type**.

	**Obese binge-type**	**Obese non-binge type**
Estimated 24-h energy requirements^a^	2547.5 (51.2)	2432.9 (60.1)
Laboratory total energy intake	3417.5 (192.2)	2590.7 (143.8)
Free-living total energy intake	2697.5 (185.9)	2329.0 (95.5)

a Estimated 24-h energy requirements were calculated using estimated resting metabolic rate multiplied by PAL score for self-reported physical activity levels.

### Effect of trait binge eating on overconsumption, macronutrient choice, and snack food intake

#### Assessment of overconsumption

To determine whether there was evidence of overconsumption in O-B and O-NB, total energy intake assessed under laboratory and free-living conditions were compared to participants' estimated 24-h energy requirements (see Table 2). Firstly, there were no differences between the estimated 24-h energy requirements of O-B and O-NB [*t*_(22)_ = 1.45, *p* = 0.16]. Under laboratory conditions, O-B consumed significantly more than their estimated 24-h energy requirements [*t*_(11)_ = 4.31, *p* < 0.001] whereas there was no difference between total energy intake and 24-h estimated energy requirements in O-NB [*t*_(11)_ = 1.22, *p* = 0.25]. Under free-living conditions, neither O-B nor O-NB consumed more than their estimated 24-h energy requirements [*t*_(11)_ = 0.887, *p* = 0.39; *t*_(11)_ = 1.05, *p* = 0.32, respectively].

#### Snack food choice and intake

The effect of trait binge eating on snack food choice and intake in the laboratory and free-living based measures of energy intake were analyzed for overall snack food energy intake, energy intake according to the taste of the snack foods (sweet or savory) and energy intake according to the taste and fat content (high or low) of the snack foods.

**Figure 1:**
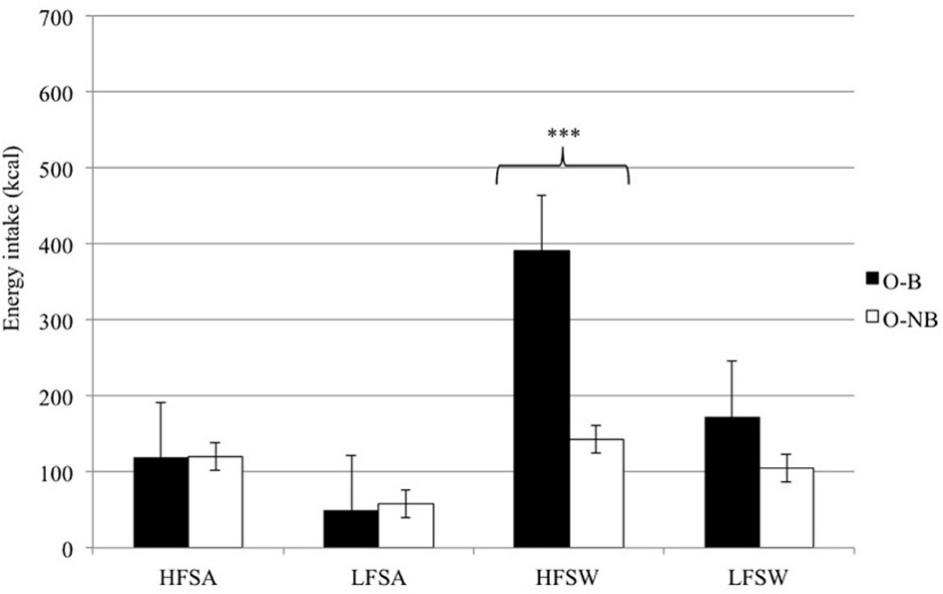
**Laboratory-based snack food choice and energy intake according to fat content and taste for O-B and O-NB**. ^***^p < 0.001.

***Laboratory-based energy intake.*** O-B consumed more energy overall from the *ad libitum* snack box compared to O-NB [*F*_(1, 22)_ = 6.92, *p* < 0.02]. An interaction between taste and binge type [*F*_(1, 22)_ = 14.43, *p* < 0.001] revealed that O-B consumed a greater number of calories from sweet foods than O-NB with both groups consuming a similar number of calories from savory foods (see Figure 1). An interaction between fat and binge type [*F*_(1, 22)_ = 4.37, *p* < 0.05] revealed that O-B consumed more energy from high fat foods although it appeared that this was accounted for by the increased intake of high-fat sweet foods as this was qualified by an interaction between fat, taste, and binge type [*F*_(1, 22)_ = 4.25, *p* < 0.05] which showed that O-B consumed 63.4% more calories from high-fat sweet foods than O-NB.

#### Free-living based energy intake

**Figure 2:**
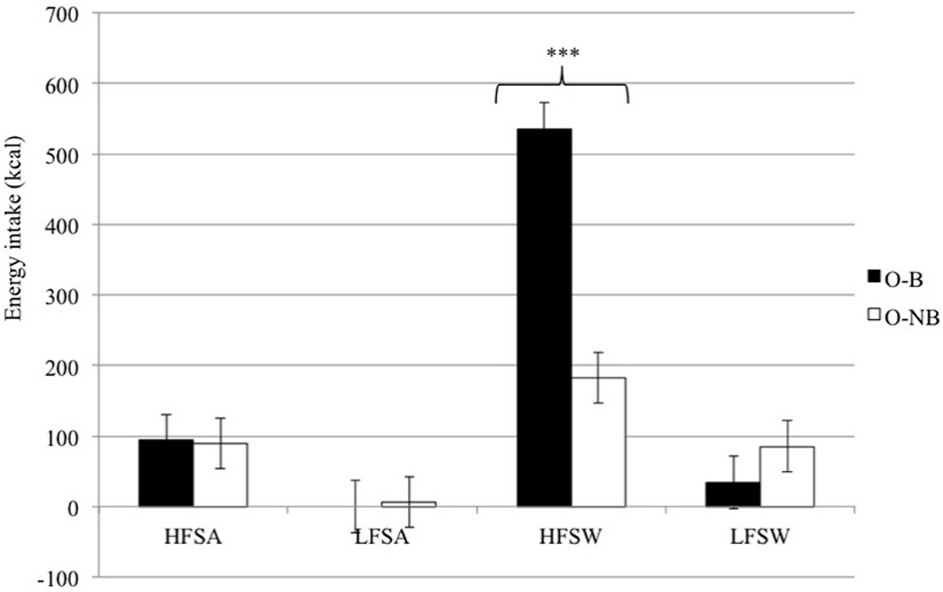
**Free-living based snack food choice and energy intake according to fat content and taste for O-B and O-NB**. ^***^p < 0.001.

O-B consumed more energy overall from snack foods in the free-living based measure compared to O-NB [*F*_(1, 22)_ = 2.52, *p* < 0.02]. An interaction between taste and binge type [*F*_(1, 22)_ = 4.23, *p* < 0.05] revealed that O-B consumed a greater number of calories from sweet foods than O-NB with both groups consuming a similar number of calories from savory foods (see Figure 2). An interaction between fat and binge type [*F*_(1, 22)_ = 9.75, *p* < 0.01] revealed that O-B consumed a greater number of calories from high-fat foods compared to O-NB foods, however, this appeared to be accounted for by the increased intake of high-fat sweet foods as there was an interaction between fat, taste, and binge type [*F*_(1, 22)_ = 4.92, *p* < 0.05] which showed that O-B consumed 71.8% more calories from high-fat sweet foods than O-NB.

### Effect of trait binge eating on food reward (LFPQ)

#### Explicit liking

Ratings of explicit liking for food were higher for both O-B and O-NB [*F*_(1, 22)_ = 46.72, *p* < 0.001] in the fasted compared to the fed state. There was a food type by binge type interaction [*F*_(3, 66)_ = 3.44, *p* < 0.02] with O-B having the greatest explicit liking ratings for high fat foods, especially high fat sweet foods when fasted (see Table 3).

**Table 3:** **Ratings (mean ± SEM) of explicit liking (mm) for O-B and O-NB for the food categories in the fasted and fed state**.

	**Fasted**		**Fed**	
	**Obese binge-type**	**Obese non-binge type**	**Obese binge-type**	**Obese non-binge type**
HFSA	66.67 (15.76)	52.96 (22.95)	34.44 (22.23)	25.77 (19.88)
LFSA	53.94 (22.95)	58.02 (13.74)	33.83 (20.59)	33.95 (11.21)
HFSW	74.52 (15.92)^**^	50.87 (21.04)^**^	47.39 (21.62)^*^	37.25 (13.09)^*^
LFSW	64.23 (11.43)	55.89 (12.26)	45.04 (15.65)	50.21 (14.08)

* p < 0.05;

** p < 0.01.

Abbreviations: HFSA, high-fat savory; LFSA, low-fat savory; HFSW, high-fat sweet; LFSW, low-fat sweet.

#### Implicit wanting

Implicit wanting for food was greater in a fasted state compared to a fed state for both O-B and O-NB [*F*_(1, 22)_ = 15.94, *p* < 0.001] but a condition by binge type interaction revealed that the O-B had enhanced implicit wanting in the fed condition compared to the O-NB [*F*_(1, 22)_ = 13.25, *p* < 0.001]. An interaction between food type and binge type revealed that O-B had greater implicit wanting for both types of sweet foods, especially high fat sweet whereas O-NB had greater implicit wanting for both low fat food categories [*F*_(3, 66)_ = 5.11, *p* < 0.01] (see Table 4).

**Table 4:** **Mean ± SEM implicit wanting (D-RT) for the obese binge-type and the obese non-binge type for the food categories in the fasted and fed state**.

	**Fasted**		**Fed**	
	**Obese binge-type**	**Obese non-binge type**	**Obese binge-type**	**Obese non-binge type**
HFSA	0.138 (0.688)	0.700 (0.815)	−0.775 (0.815)	−0.792 (0.689)
LFSA	−0.636 (0.493)	0.120 (0.585)	−0.121 (0.752)	−0.067 (0.867)
HFSW	0.228 (0.405)^**^	−0.475 (0.438)^**^	0.667 (0.272)^*^	0.381 (0.334)^*^
LFSW	−0.058 (0.321)	−0.371 (0.504)	0.443 (0.377)	0.477 (0.341)

* < 0.05;

** p < 0.01.

Abbreviations: HFSA, high-fat savory; LFSA, low-fat savory; HFSW, high-fat sweet; LFSW, low-fat sweet.

### Effect of trait binge eating on cravings for food

O-B scored significantly higher on the Craving Intensity [*t*_(20)_ = 2.35, *p* < 0.05] and Craving for Sweet Food [*t*_(20)_ = 2.86, *p* < 0.01] subscales of the COEQ, and lower on the Mood subscale [*t*_(21)_ = 2.26, *p* < 0.05] compared to O-NB. There were no differences between O-B and O-NB on the Craving for Savory Food [*t*_(20)_ = 1.71, *p* = 0.10] or Feelings of Fullness [*t*_(20)_ = 1.63, *p* = 0.12] subscales of the COEQ.

## Discussion

The present study examined food intake and food choice in the laboratory and under free living conditions over two 24-h periods in order to address two aims: (1) to examine the concordance between laboratory-based and free-living based measures of eating behavior, (2) determine whether the previously identified processes and behaviors associated with the trait binge eating behavioral subtype of obesity were evident in free-living eating behavior. We hypothesized that greater levels of binge eating would be associated with greater energy intake and a preference for high-fat sweet foods under both laboratory and free-living conditions. In addition, in line with our previous findings, we hypothesized that “binge-types” would have greater liking and wanting for high-fat sweet foods compared to “non-binge types.”

There was a significant association between overall energy consumed under laboratory and free-living conditions supporting the validity of laboratory-based test meal methodologies. Consistent with our hypothesis, we found that O-B consumed a greater amount of energy in the laboratory compared to O-NB. In addition, we found evidence to support that O-B had over-consumed in the laboratory-based measure of energy intake as they ate significantly more than their estimated energy requirements. However, there were no differences between O-B and O-NB in total energy consumed in the free-living based measure of energy intake, with neither group consuming significantly above their estimated energy need. The disparity in findings between the two measures of energy intake may have arisen for several reasons. Firstly, the current study provided all test meals in *ad libitum* quantities and within each test meal there were several different items that participants could choose from. Previous research has shown that providing participants with a variety of food stimulates intake by delaying the development of satiation (Hetherington et al., 2006). While it can be argued that compared to the free-living environment, the variety of food items provided under laboratory conditions is limited, the immediate availability (prepared and ready to eat) of a variety of food items available under these conditions may be conducive to increasing consumption above normal levels, especially in those susceptible to overeating. Secondly, with regards to the obese binge-type group in particular, it may be that the increased intake observed in the laboratory-based period had an impact on food intake the following day, reducing it below normal levels. In order to address this, future research could include two or three 24-h dietary recalls, independent from the laboratory session, in order to obtain a more representative average of free-living intake. Thirdly, dietary recall procedures are vulnerable to high rates of underreporting partly due to difficulties in remembering dietary details and partly due to embarrassment regarding how much was eaten. Therefore, the free-living energy intake measure may not be an accurate reflection of total energy consumed (Moshfegh et al., 2008).

Consistent with previous reports (Davis et al., 2009; Finlayson et al., 2011; Dalton et al., 2013), we found that greater levels of binge eating were associated with increased intake of and preference for high fat sweet foods. We extended our previous findings by showing that this preference for high fat sweet foods applied to eating behavior in the natural setting, with O-B exhibiting a greater preference for sweet foods under both laboratory and free-living conditions. Furthermore, these findings were supported by greater laboratory-based measures of food reward and food cravings. Specifically, we found that O-B had enhanced liking for foods in the behavioral task, in particular high fat foods. We also found an enhanced implicit wanting for high-fat sweet foods in O-B compared to O-NB, especially during a fasted state. The finding that O-B had enhanced liking for food and enhanced wanting for high-fat sweet food is consistent with previous research (Davis et al., 2009, 2011; Finlayson et al., 2011; Dalton et al., 2013).

In addition to examining food intake and food choice we also investigated whether there were any self-reported differences for the preceding 7 days using the Mood subscale from the Control of Eating Questionnaire (COEQ; Hill et al., 1991). We found that O-B reported experiencing overall lower positive mood compared to O-NB. This finding is in line with previous research that has shown individuals with subclinical binge eating behavior tend to report experiencing lower moods (Greeno et al., 2000; Wegner et al., 2002; Goldschmidt et al., 2011, 2012). Greeno et al. (2000) found that individuals with binge eating tendencies experienced lower positive mood compared to those who did not engage in binge eating. Interestingly, some evidence (Wegner et al., 2002) suggests that the experience of low mood may be a characteristic of individuals with the tendency to binge eat that is independent from the behavior itself.

Although the present study carried a number of strengths, there were also some limitations to consider. Firstly, although participants were presented with a large quantity of food and were informed that they could request more of any item, we cannot rule out that some participants could have felt inhibited and may have eaten more if provided with larger initial servings. Secondly, the findings from the 24-h dietary recall procedure may not be representative of habitual eating behavior as only one measure was taken. The procedure would be more representative if conducted on at least three different occasions in line with previous research (Moshfegh et al., 2008; Epstein et al., 2011; Stote et al., 2011). Thirdly, participants' evening intake on Day 1 was assessed using dietary recall rather than being objectively measured under laboratory conditions, which may have an impact on the validity of these results. Finally, the sample consisted of females recruited from or near a University campus; therefore, the applicability of the findings to the wider population may be limited. In addition, the sample size in the current study may pose a limitation to the interpretation of the results with regards to the smaller effect sizes in the trait binge eating subtype.

In summary, while laboratory-based and free-living based measures were not identical with regards to total energy consumed, they did provide a coherent view of trait binge eating with greater energy intake of snack foods, and a preference for high-fat sweet foods observed under both laboratory and free-living conditions. These findings were in line with previous reports (Finlayson et al., 2011; Dalton et al., 2013) and were supported by increased scores of liking, wanting, and food cravings for high-fat sweet foods. Identification of traits and behaviors that increase an individual's susceptibility to overconsumption may help to develop more tailored strategies for the prevention and treatment of weight gain. The distinct pattern of preference for sweet foods observed in the binge type obese carries implications for the control of food intake in individuals susceptible to overeating (Blundell and Finlayson, 2008). Finally, interventions that aim to reduce psychological traits associated with overeating (where weight loss is not the primary outcome) could supplement and enhance the success of existing weight loss strategies.

### Conflict of interest statement

The authors declare that the research was conducted in the absence of any commercial or financial relationships that could be construed as a potential conflict of interest.
